# Prognostic Impact of Tumor Extension in Patients With Advanced Temporal Bone Squamous Cell Carcinoma

**DOI:** 10.3389/fonc.2020.01229

**Published:** 2020-08-07

**Authors:** Noritaka Komune, Masaru Miyazaki, Kuniaki Sato, Koji Sagiyama, Akio Hiwatashi, Takahiro Hongo, Kensuke Koike, Teppei Noda, Ryutaro Uchi, Ryunosuke Kogo, Nana Akagi Tsuchihashi, Shogo Masuda, Takashi Nakagawa

**Affiliations:** ^1^Department of Otorhinolaryngology, Graduate School of Medical Sciences, Kyushu University, Fukuoka, Japan; ^2^Department of Otorhinolaryngology, Fukuoka University Hospital and School of Medicine, Fukuoka, Japan; ^3^Department of Clinical Radiology, Graduate School of Medical Sciences, Kyushu University, Fukuoka, Japan

**Keywords:** squamous cell carcinoma, temporal bone, middle ear, external auditory canal, prognosis factor

## Abstract

**Objective:** The extreme rarity of temporal bone squamous cell carcinoma (TB-SCC) has delayed the accumulation of high-quality clinical evidence. Our objective here was to explore anatomical factors associated with the prognosis of T4 TB-SCC cases.

**Study Design:** Case series with chart review.

**Setting:** Two academic tertiary care medical centers.

**Subjects and Methods:** The medical records of all TB-SCC cases were retrospectively reviewed in two institutions. The resulting data set contained 30 cases of primary T4 cancer eligible for initial definitive (curative) treatment. Disease-specific survival was calculated according to the Kaplan–Meier method. Cox proportional hazards model was used to identify anatomical prognosis factors.

**Results:** The disease-specific 5-years survival rate of 30 cases of T4 TB-SCC was 53.9%. The tumor invasion to the pterygoid muscle, posterior fossa dura, and sigmoid sinus and destruction of the ossicles were associated with poor prognosis in univariate analysis. The multivariate analysis reveals that the invasion of the ossicles, posterior fossa dura, and sigmoid sinus is an independent prognostic factor [hazard ratio (HR): 4.528 (95% CI: 1.161–17.658), p = 0.030; HR: 5.135 (95% CI: 1.616–16.315), p = 0.006; HR: 4.292 (95% CI: 1.385–13.303), p = 0.012]. The invasion of the carotid canal, petrous apex, middle fossa dura, otic capsule, pterygoid muscle, and middle ear had a high HR (HR > 2). The more invaded anatomical factors present in patients resulted in a poorer patient disease-specific prognosis, with a statistically significant difference.

**Conclusions:** Assessing which anatomical structures are susceptible to invasion by tumors may be important for predicting TB-SCC patient prognosis and selecting appropriate treatment planning, especially surgical intervention. In addition to previously reported factors, the destruction of the ossicles in the middle ear cavity can be an anatomical prognosis factor.

## Introduction

Malignant neoplasms of the temporal bone are extraordinarily rare and account for <0.2% of all head and neck malignancies (1). Squamous cell carcinoma (SCC) is the most common form of temporal bone malignant malignancies, followed by adenoid cystic, metastatic tumor, and mucoepidermoid carcinomas, among others. The low occurrence of temporal bone SCC (TB-SCC) has limited the amount of available data from both clinical and basic research. The Pittsburgh classification system is a globally popular staging system for TB-SCC, especially for the external auditory canal (EAC) carcinoma (1, 2). However, this scheme may not accurately reflect tumor extension and lumps resectable and unresectable tumors in the same category: T4. In the eight edition of the American Joint Committee on Cancer (AJCC) staging system, TB-SCC is not classified into a unique category and is considered a cutaneous SCC (3). Pensak et al. (4) reported clinical data from carcinomas with temporal origins using the University of Cincinnati Medical Center grading system for temporal bone tumors, which roughly considers anatomical tumor extension. In 1997, Kishimoto et al. (5) proposed a unique staging system that reflects the direction of tumor extension and invaded anatomical structures. However, the description is written in Japanese, and the system is thus not popular on a global scale. Kishimoto et al.'s (5) classification system, as shown in Table 1, does not reflect the impact of facial paralysis and the thickness of soft tissue invasion. Each classification system used previously has pros and cons (1–5). Until now, there has been no classification system that correlates invaded structures with the prognosis for patients with this malignancy. Therefore, based on these reports, the current global classifications, including the modified Pittsburgh classification, need reevaluating.

**Table 1:** Previously reported classifications of temporal bone squamous cell carcinomas.

**Clasification**							
**T**		**AJCC 8**	**(****6****)**	**(****1****)**	**(****5****)**	**T**	**(****4****)**
**I**		Tumor smaller than 2 cm in greatest dimension	Tumor limited to site of origin, i.e. with no facial nerve paralysis and no bone destruction	Tumor limited to the external auditory canal without bony erosion or evidence of soft tissue extension	Tumor limited to the external auditory canal without bony erosion	**I**	Tumor in a single site, 1 cm or less in size
**II**		Tumor 2 cm or larger, but smaller than 4 cm in greatest dimension	Tumor extending beyond the site of origin indicated by facial paralysis or radiological evidence of bone destruction, but no extension beyond the organ of origin	Tumor with limited external auditory canal bony erosion (not full thickness) or limited (< 0.5 cm) soft tissue involvement	Tumor with limited external auditory canal bony erosion (not full thickness) or invasion of auricle	**II**	Tumor in a single site, >1 cm in size
**III**		Tumor 4 cm or larger in maximum dimension, minor bone erosion, perineural invasion or deep invasion	Clinical or radiological evidence of extension to surrounding structures (dura, base of the skull, parotid gland, temporomandibular joint, etc.)	Tumor eroding the osseous external auditory canal (full thickness) with limited (< 0.5 cm) soft tissue involvement, or tumor involving middle ear and/or mastoid	Tumor extends beyond the external auditory bony canal: mastoid cavity, tympanic cavity, fallopian canal, ossicles	**III**	Transannular tumor extension
**IV**	**IVa**	Tumor with gross cortical bone/marrow invasion	(Tx:) Patients with insufficient data for classification, including patients previously seen and treated elsewhee	Tumor eroding the cochlea, petrous apex, medical wall of the middle ear, carotid canal, jugular foramen or dura, or with extensive soft tissue involvement (> 0.5 cm), such as involvement of temporomandibular joint or styloid process, or evidence of fasical paresis	Tumor involves the mandibular fossa, sigmoid sinus, jugular bulb, eustachian tube, petrous apex, inner ear, framen ovale. foramen lacerum, infratemporal fossa, carotid canal, parotid gland, temporal muscle, skin around auricle, etc.	**IV**	Mastoid or petrous air-cell invasion
	**IVb**	Tumor with skull base invasion and/or skull base foramen involvemen			Intracranial extension including dural invasion	**V**	Periauricular or contiguous extension (extratemporal)
						**VI**	Neck adenopathy, distant anatomic site, or infratemporal fossa extension

The complex, intricate structure of the temporal bone is due to its close associations with vital organs. Its anatomical structures, either in whole or in part, include the internal carotid artery (ICA), otic capsule, sigmoid sinus, jugular bulb, superior and inferior petrosal sinuses, internal auditory canal, the trigeminal and lower cranial nerves, and the eustachian tube. The temporal bone is surrounded by the dura of the middle and posterior cranial fossae, infratemporal fossa, temporomandibular joint (TMJ), parotid gland, and parapharyngeal space. There are few studies examining the anatomical factors affecting the prognosis of advanced TB-SCC (1, 7). In this study, we examined the preoperative radiological findings of contrast computed tomography (CT) and magnetic resonance imaging (MRI) to reveal these factors in cases with T4 advanced TB-SCC.

## Materials and Methods

### Ethics Statement

Our study was conducted with the approval of the ethics review committee of both Kyushu University Hospital (permit no. 29–43) and Fukuoka University Hospital (permit no. 2017M091).

### Patients and Preoperative Staging

In this study, the tumor stages of cases were defined using the modified Pittsburgh classification. Clinical outcomes were analyzed for applicable patients treated at the Department of Otorhinolaryngology Head and Neck Surgery of two tertiary referral centers (Kyushu and Fukuoka University Hospitals) between April 2006 and December 2017. T4 cases that underwent definitive treatment and follow-up for at least 2 years after treatment were selected for this study. We used the Eastern Cooperative Oncology Group (ECOG) performance status scale to estimate the patients' physical functioning. All cases showed good performance status (PS), defined as PS 0–2 on the ECOG scale. Cases not undergoing treatment with sufficient intensity [either due to poor PS (with poor performance defined as PS 3–4) or patient refusal] were excluded. The final cases sampled for our retrospective cohort study consisted of 30 patients specifically diagnosed with T4 TB-SCC.

Prior to surgery, all cases underwent contrast-enhanced CT and MRI. Temporal bone CT images were obtained using a 64-detector-row CT scanner (Aquilion 64, Toshiba Medical Systems, Otawara, Japan) or a 320-detector-row CT scanner (Aquilion One, Toshiba Medical Systems) with 0.5-mm collimation and a 512 × 512 matrix after an infusion of 2 ml/kg of a non-ionic iodinated contrast agent. Transverse scans were acquired in a plane parallel to the orbitomeatal plane in the helical mode with 120 kV, 250 mAs, 0.5-mm section thickness and overlap 0.3 mm with its adjacent slice, beam pitch 0.625, scan field of view (FOV) 240 mm, and display FOV 80 mm. MRI scans were performed on a 1.5-Tesla imaging unit (Achieva, Philips Medical Systems, Best, The Netherlands) or a 3-Tesla unit (Ingenia, Philips Medical Systems) with a 15-channel head array receiving coil for sensitivity encoding (SENSE) parallel imaging. Transverse T2-weighted images (TR/TE = 3,500/80 ms, FA = 90, 12 slices, slice thickness/gap = 2/1 mm, FOV = 170 mm, matrix = 304 × 238, NSA = 2), coronal T2-weighted images (30 slices, slice thickness/gap = 3/1 mm, FOV = 240 mm, matrix = 320 × 242, NSA = 2), and transverse T1-weighted images (TR/TE = 550/15 ms, FA = 90, 12 slices, slice thickness/gap = 2/1 mm, FOV = 170 mm, matrix = 224 × 181, NSA = 2) were acquired, followed by contrast-enhanced transverse T1-weighted images after intravenous injection of 0.1 mmol/kg of gadolinium-contrast agent. In addition, three-dimensional (3D) T1-weighted images (3D-FFE, TR/TE = 18/3.7 ms, FOV = 180 mm, matrix = 512 × 512, reconstruction thickness = 1 mm, NSA = 2) were acquired. 18-Fluorodeoxyglucose positron emission tomography (^18^FDG PET)-CT with 4.0 MBq/kg of ^18^FDG was performed to check for distant metastasis. At least two otorhinolaryngologists and two head and neck radiologists jointly assessed the extent of local progression and bone damage, confirming the T stage progression.

**Figure 1:**
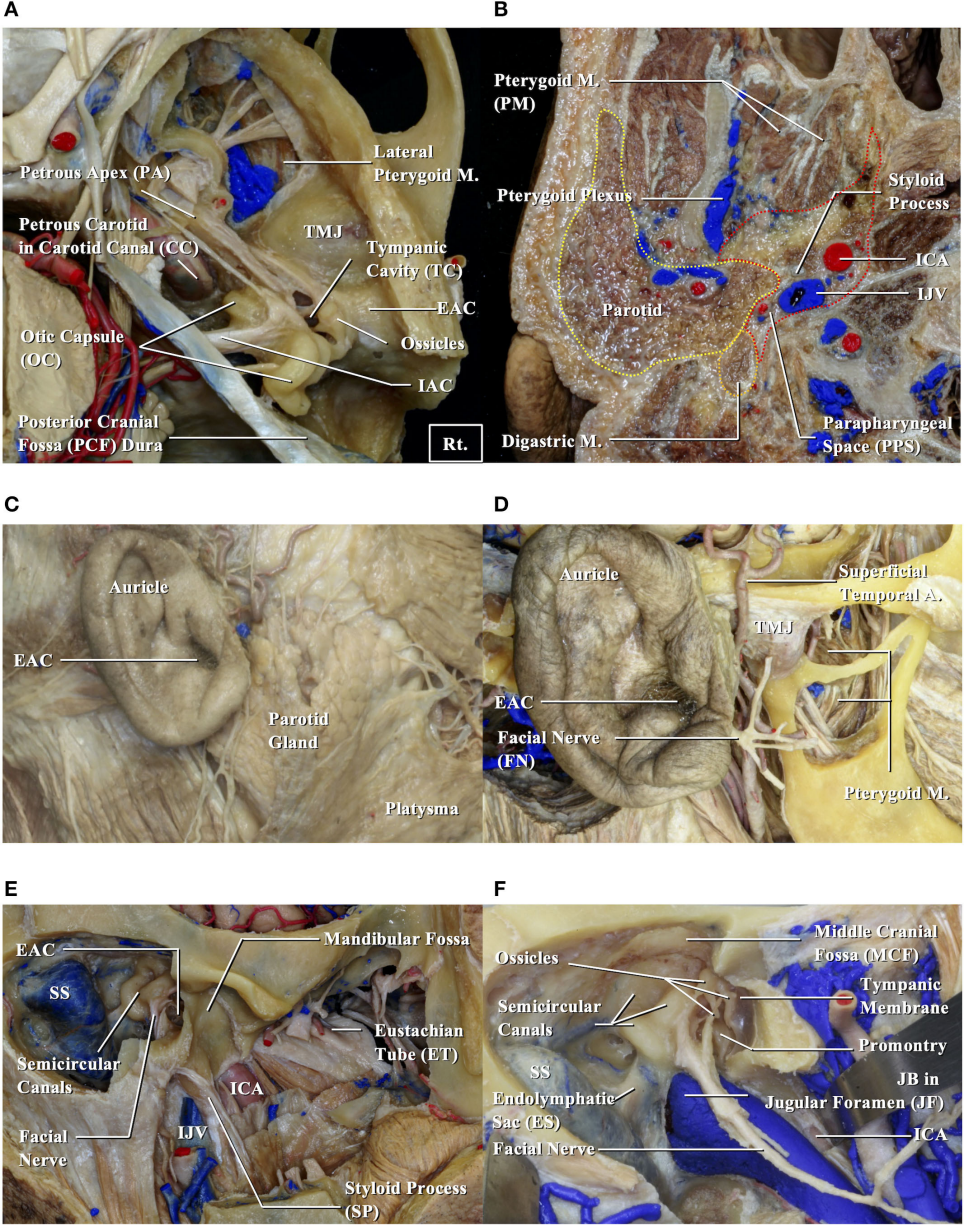
Anatomical structures related to EAC squamous cell carcinomas. EAC, external auditory canal; ICA, internal carotid artery; IJV, internal jugular vein; JB, jugular bulb; M, muscle; SS, sigmoid sinus; TMJ, temporomandibular joint. **(A,B)** Cadavers are dissected from above **(A)** and below **(B)**. **(C)** Structures around the auricle are exposed. **(D)** Structures around the temporomandibular joint are exposed. **(E)** Structures related to the bony part of EAC are exposed. **(F)** Relationship between the middle ear and surrounding structures is shown.

To examine anatomical prognosis factors, we examined 17 structures in the temporal bone among T4 TB-SCC patients, including the tympanic cavity, ossicles (the destruction of the ossicles in the middle ear), eustachian tube (the infiltration of the tympanic orifice in the eustachian tube), parotid gland, middle and posterior cranial fossae, petrous apex, carotid canal, jugular foramen (fossa), otic capsule, facial nerves (facial nerve paralysis), the TMJ, pterygoid muscle, parapharyngeal space, sigmoid sinus, endolymphatic sac, and styloid process (Figure 1).

### Treatment Strategy

Our basic policy was to perform surgery for all resectable cases (T1–T4) that consented to treatment. Among the advanced T4 cases, induction chemotherapy or preoperative chemoradiation therapy (CRT) were selected to reduce tumor size if the tumor could not initially be treated with lateral temporal bone resection (LTBR). Radiotherapy was administered 5 days per week (1.6–2.0 Gy/fraction for a total dose of 30–40 Gy). Patients were scheduled for surgery if the tumor shrank to a resectable size as a result. If the tumor shrank sufficiently to be treated with LTBR, we performed LTBR rather than subtotal temporal bone resection (STBR). Inoperable cases were given curative CRT that targeted the primary tumor focus and lymph nodes. We selected a total dose of 60–70 Gy, including boost doses. Surgical intervention was considered if lesions shrank to a resectable size during CRT. Curative resections were followed by adjuvant CRT if a positive margin was confirmed.

For the first 4 weeks of radiation therapy (RT) (4 weeks, 1 week rest), patients were given intravenous 5-fluorouracil (5-FU; 250 mg/day) or oral S-1, a fluoropyrimidine anticancer drug (Taiho, Tokyo, Japan; tegafur equivalent = 65 mg/m^2^) to potentiate the course's effects. Since 2015, instead of S-1, patients received triweekly cisplatin (CDDP: 100 mg/m^2^, every 3 weeks, two to three cycles), a standard treatment for other head and neck SCCs (HNSCCs). We selected a docetaxel, cisplatin, and fluorouracil (TPF) regimen for patients as induction chemotherapy: 5-FU (600 mg/m^2^; days 1–5) + CDDP (60 mg/m^2^/day; day 1) + docetaxel (DOC: 60 mg/m^2^; day 1) every 3 weeks for one to two cycles.

### Statistical Analysis

The relationship between survival and the anatomical factors was examined using a univariate Cox proportional hazards model. We performed a multivariable analysis after adjusting for gender and age (≤ 65) as covariates. Survival rates were calculated using the Kaplan–Meier method and compared using the log-rank test. All estimates below are the disease-specific 5-years survival (DSS) rate unless otherwise noted. The DSS rate was the same as the overall survival rate during our research period. JMP 6.1 was used for statistical analysis, and p < 0.05 indicated statistical significance.

## Results

**Figure 2:**
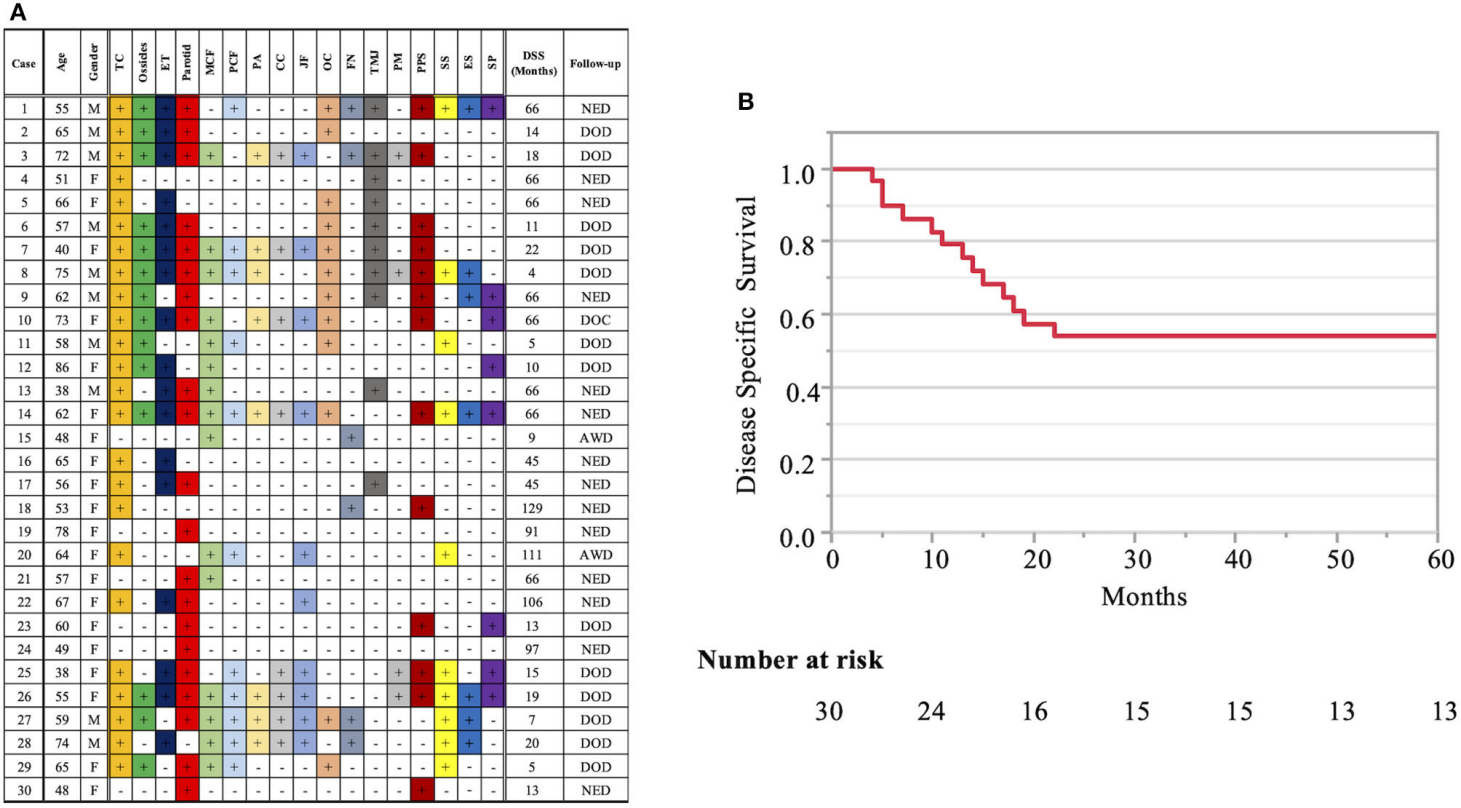
Detailed information on the invaded structures (17 landmarks) **(A)** and disease-specific 5-years survival curve in all T4 cases **(B)**. Anatomical factors examined include the tympanic cavity (TC), ossicles (the destruction of the ossicles in the middle ear), eustachian tube (ET; the infiltration of the tympanic orifice of the eustachian tube), parotid gland, middle and posterior cranial fossae (MCF and PCF), petrous apex (PA), carotid canal (CC), jugular foramen (fossa) (JF), otic capsule (OC), facial nerve (FN; facial nerve paralysis), temporomandibular joint (TMJ), pterygoid muscle (PM), parapharyngeal space (PPS), sigmoid sinus (SS), endolymphatic sac (ES), and styloid process (SP).

**Figure 3:**
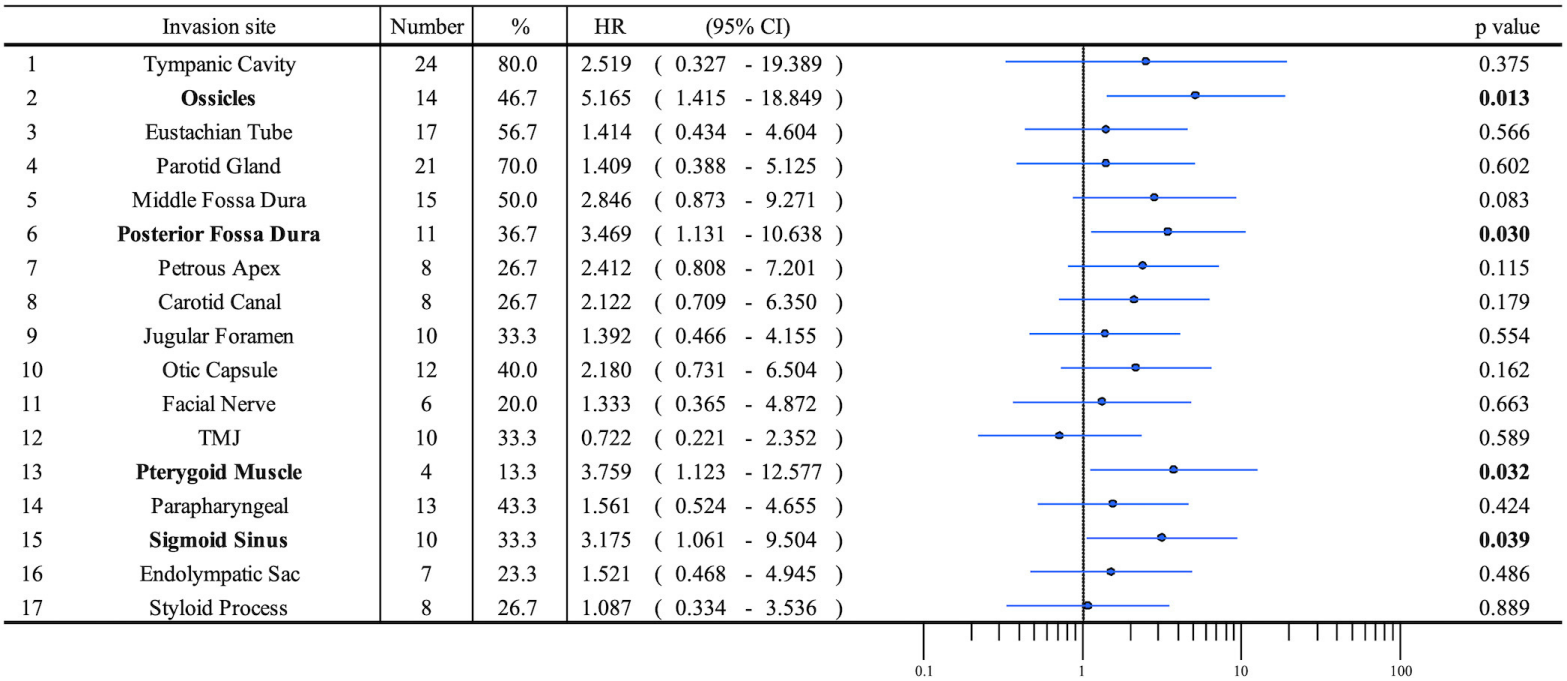
Anatomical factors predicting survival (univariate analysis) using a forest plot.

Patient profiles are shown in Table 2. Figure 2A shows information on the invaded structures for all cases, and Figure 2B shows the DSS for all cases (53.94%). We then examined the relationship between invasion of the 17 anatomical landmarks and the prognosis of the patient. Univariate analysis for the anatomical prognosis factors identified invasion of the pterygoid muscle and posterior cranial dura and destruction of the ossicles and sigmoid sinus as significant predictors of poor prognosis (Figure 3). Extension into the otic capsule, petrous apex, middle cranial fossa dura, and carotid canal showed a high hazard ratio (HR > 2) but was not statistically significant (p ≥ 0.05; Figure 3).

**Table 2:** Patient profiles.

	**N**	**%**
**AGE GROUPS**		
<65	19	63
65 ≤	11	37
**GENDER**		
Male	10	67
Female	20	33
**LYMPHNODE METASTASIS**		
+	9	30
−	21	70
**DISTANT METASTASIS**		
+	0	0
−	30	100
**PATHOLOGICAL FEATURE**		
Poor. diff.	2	7
Mod. diff.	5	17
Well diff.	20	67
SCC with clear cell change or mucoepidermoid carcinoma	1	3
SCC with sarcomatoid change	1	3
Unknown	1	3
**TREATMENT**		
(C)RT+Surgery	14	47
Surgery	1	3
CRT only	10	33
iaChemo+CRT	3	10
Other	2	7
**SURGICAL INTERVENTION**		
LTBR	7	23
STBR	8	27
None	15	50

**Figure 4:**
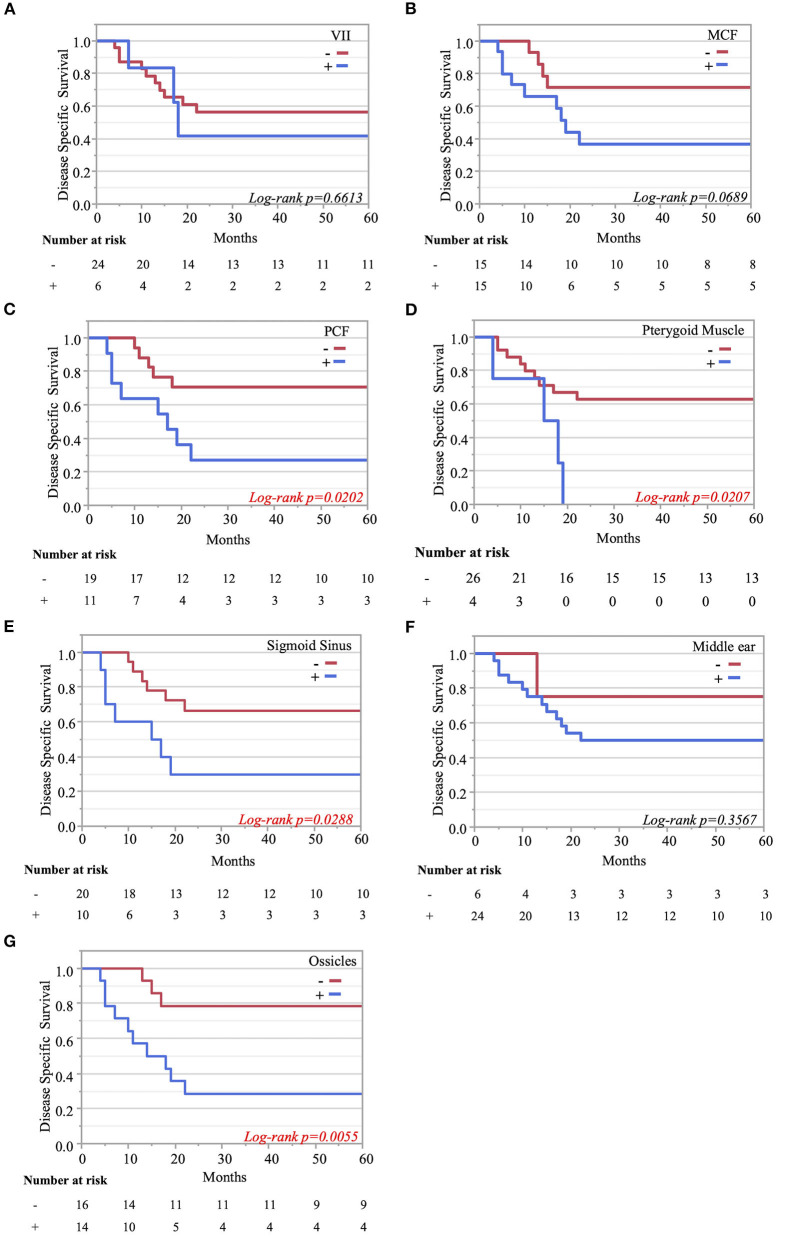
Kaplan–Meier curves. Disease-specific 5-years survival curves according to tumor invasion of the facial nerve **(A)**, middle cranial fossa **(B)**, posterior cranial fossa **(C)**, pterygoid muscle **(D)**, sigmoid sinus **(E)**, middle ear **(F)**, and ossicle destruction **(G)**.

**Figure 5:**
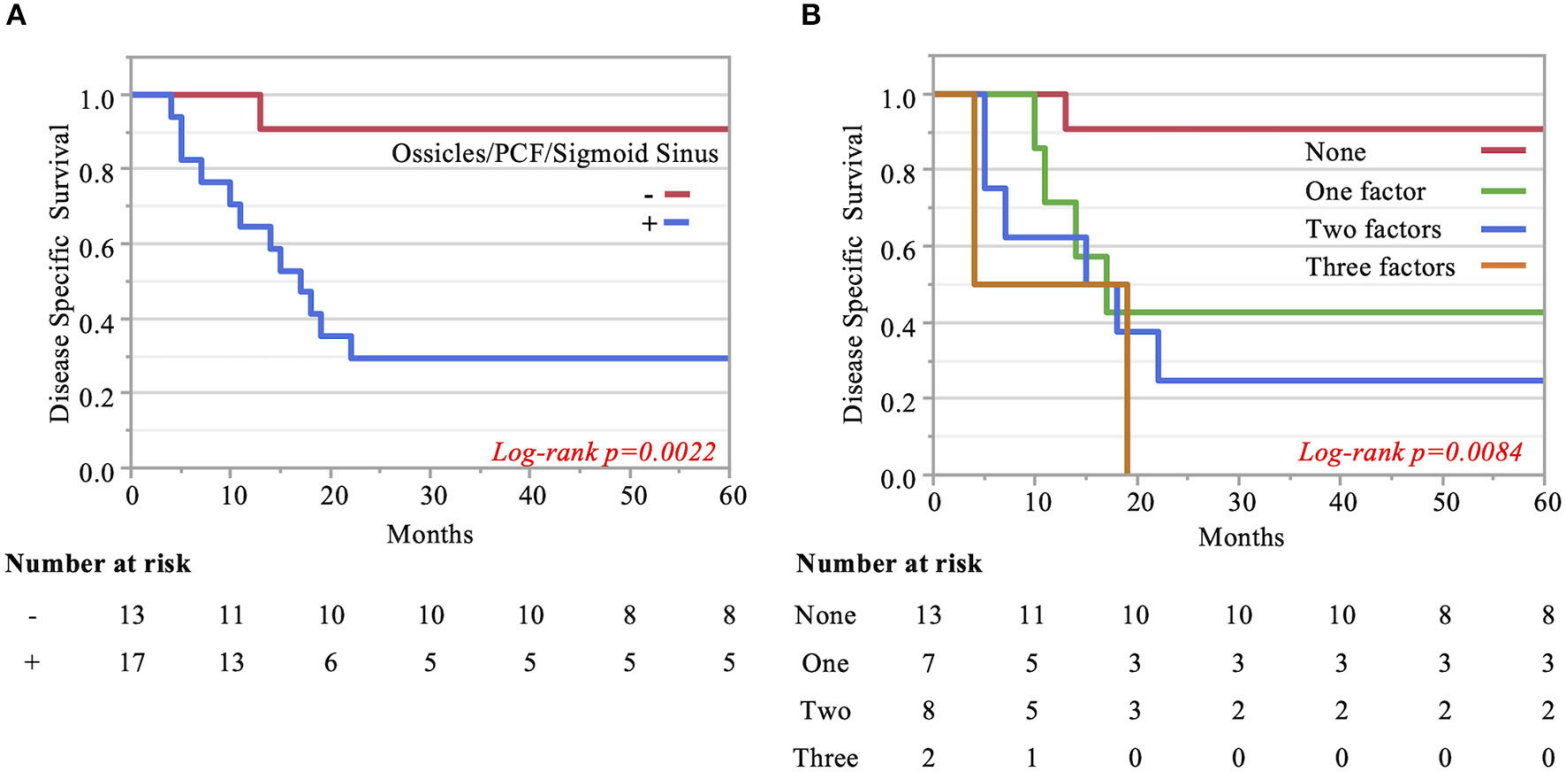
Kaplan–Meier curves. **(A)** Two groups are compared: cases with invasion of at least one of the three significant structures and cases without invasion of any of these three factors. **(B)** Pterygoid muscle invasion, ossicle destruction, and invasion of the posterior fossa dura/sigmoid sinus are regarded as an anterior/inferior invasion marker, medial invasion marker, and posterior invasion markers, respectively. The impact of the number of invasion markers was analyzed among all three categories. The more markers, the poorer the patient disease-specific prognosis, which was statistically significant.

The Kaplan–Meier curves showed that cases with invaded ossicles, sigmoid sinus, pterygoid muscle, or posterior fossa dura had a significantly worse prognosis than cases without the invasion of these structures (p = 0.0055, 0.0288, 0.0207, and 0.0202, respectively; Figure 4). The invasion of the tympanic cavity, middle fossa dura, petrous apex, carotid canal, and otic capsule was associated with decreased survival rates among the T4 cases, although this did not reach statistical significance (Figure 4). Results from the multivariate analysis for prognosis factors influencing the DSS rate among the T4 cases for TB-SCC are shown in Table 3. Invasion of the ossicles [HR: 4.528 (95% CI: 1.161–17.658), p = 0.030], posterior fossa dura [HR: 5.135 (95% CI: 1.616–16.315), p = 0.006], and sigmoid sinus [HR: 4.292 (95% CI: 1.385–13.303), p = 0.012] were independent prognostic factors. We divided cases into two groups: cases with invasion of at least one of these three structures and cases without invasion of any of these three structures. The Kaplan–Meier curves show that cases without invasion of any of the three structures had a significantly improved DSS rate compared to cases with invasion of any one of the three structures (90.91 vs. 29.41%, respectively, p = 0.0022; Figure 5A).

**Table 3:** Anatomical factors predicting survival (multivariate analysis).

**Invasion site**	**Multivariate analysis**				
	**HR**	**p-value**	**95% CI**		
Ossicles	4.528	**0.030**	1.161	-	17.658
Posterior fossa dura	5.135	**0.006**	1.616	-	16.315
Pterygoid muscle	2.902	0.099	0.819	-	10.284
Sigmoid sinus	4.292	**0.012**	1.385	-	13.303

Finally, we examined the impact of the number of invaded structures. Univariate and multivariate analyses for prognostic factors influencing DSS rate showed that pterygoid muscle invasion could be regarded as an anterior/inferior invasion marker, ossicle invasion as a medial invasion marker, and posterior fossa dura or sigmoid sinus invasion as a posterior invasion marker. We found that the more factors present in patients resulted in a poorer patient disease-specific prognosis, with a statistically significant difference (p = 0.008). The DSS rates were as follows: 90.9% (no factor), 42.9% (one factor), 25% (two factors), and 0.0% (three factors) (Figure 5B).

## Discussion

The extreme rarity of TB-SCC causes a delay in building the high-quality evidence for its treatment. While no standard protocol has been established for its treatment, margin-negative resection is widely considered a viable treatment strategy for TB-SCC. Our treatment strategy for T4 cases included the induction chemotherapy or preoperative CRT to achieve *en bloc* and margin-negative resection. A few studies reported the effectiveness of preoperative CRT to achieve a high control rate after *en bloc* resection (8, 9). To control the residual lesions in cases with positive surgical margins, postoperative RT is reportedly an excellent strategy, (1, 4, 9, 10) as well as findings of the EORTC 22931 and RTOG 9501 trials in other HNSCCs (11, 12).

To examine the tumor extension accurately is necessary to achieve the margin-negative resection. A preoperative analysis of both contrast-enhanced MRI and CT is mandatory for determining the tumor extension of TB-SCC. High-resolution CT of the temporal bone is sensitive to bone erosion and can help define the extent of the mass using contrast enhancement (13). It is also essential to detect bone destruction (geographic, moth-eaten, or permeative pattern) in areas such as the jugular fossa, carotid canal, posterior and middle cranial bases, tegmen, TMJ, and petrous apex. Avascular labyrinthine bone is reported to be relatively unaffected in temporal bone malignancy (14). Bone resorption in certain areas should be suspected as signs of tumor invasion, which is often difficult to distinguish from inflammatory changes. Thus, once malignancy is suspected, a biopsy should be performed immediately. MRI is more effective for demonstrating associated soft tissue infiltration. TB-SCC often shows soft tissue invasion without any clear demarcation. Furthermore, on CT, it is difficult to distinguish between mucosal thickening and tumor in the middle ear without bone erosion. MRI before and after contrast enhancement provides excellent delineation of soft tissue tumor margins and infratemporal fossa and parapharyngeal space infiltration. Sagittal and coronal planes are helpful for demonstrating the contiguous involvement of the surrounding area. Most of the lesions appeared iso-intense on the T1-weighted image and heterogeneously hyper-intense on the T2-weighted image (15). Heterogeneous enhancement can be found due to necrosis. Contrast-enhanced MRI is the best sequence for identifying dural invasion, which shows thickening of the dura and nodular contrast enhancement (15).

The relationship between tumor extension and patient prognosis is important to consider with surgical intervention for advanced TB-SCC. Several classification systems for TB-SCC have been designed (Table 1), each with their own pros and cons. The field would benefit from a modified Pittsburgh staging system that aligns with surgical procedures by more explicitly considering extension range. Other HNSCC, laryngeal, or pharyngeal carcinoma use a staging system that reflects the tumor extension. For TB-SCC, establishing such a staging system would help to standardize the treatment strategies. The rarity of this tumor type makes it difficult to examine anatomical prognosis factors, so we can only discuss these factors using a few cases and limited clinical experience. Furthermore, many investigators have grouped tumors with different histologies in the same analysis and used origin sites other than the temporal bone, such as secondary temporal bone invasion from the parotid cancer and auricle (16, 17), which make the results from these studies difficult to interpret. In this study, we focused on the analysis of advanced TB-SCC.

In the modified Pittsburgh classification system, extensive soft tissue involvement (>0.5 cm) was considered T4 (1). However, the two types of cases that can be treated with either LTBR or STBR for curative resection are placed into the same category: T4. Therefore, it is difficult to accurately predict prognosis (8). Ito et al. (18). pointed out that soft tissue involvement does not correlate with prognosis. We hypothesized that the invaded anatomical structures are more important in predicting prognosis than the thickness of the soft tissue. Identifying anatomical prognostic markers is necessary to establish an appropriate staging system. Many surgeons consider the extent of tumor invasion to be associated with prognosis. However, it is difficult to collect enough advanced T4 cases in a single center for sufficient statistical analysis. Therefore, in this study, we analyzed the relationship between tumor extent and patient DSS rate with 30 T4 TB-SCC cases from two tertiary referral centers.

The most common site of TB-SCC is the external auditory meatus. The bone and cartilage of the EAC and tympanic membrane can be a barrier to tumor diffusion. However, tumors easily cross this barrier and extend inferiorly and anteriorly through the bone–cartilage junction, the fissures of Santorini, and the foramen of Huschke (dehiscence) located anteroinferior to the osseous EAC and posteromedial to the TMJ (19). However, it is well-known that TB-SCC, representing EAC SCCs, arises from various sites and demonstrates a multidirectional pattern of growth with and without bony invasion.

If the tumor extends medially, the otic capsule, petrous apex, carotid canal, and jugular bulb may be invaded. The bone plates over many structures, such as the jugular bulb, carotid tegmen, fallopian canal, and labyrinth, are thin and hence vulnerable to tumor erosion (20). Inferior extension of the tumor results in invasion of the parapharyngeal space, including the carotid sheath, which surrounds the ICA, internal jugular vein, and lower cranial nerves. The invasion of the ICA makes curative resection impossible. Posterior extension of the tumor reaches the posterior cranial fossa dura following mastoid air cell destruction. Superior extension easily invades the middle cranial fossa dura through the middle ear due to the thin roof of the middle ear. If the origin of the SCC is the middle ear or if the EAC SCC extends into the middle ear, the tumor easily invades the middle cranial fossa dura. Previous studies show that bony invasion (18, 21, 22); facial paralysis (6, 18, 23); and invasion of the middle ear (21), dura (20, 21, 24), petrous apex (25), jugular foramen (25, 26), ICA (22), and TMJ (24) are associated with a poor outcome.

In this study, our univariate analysis showed that the invasion of the pterygoid muscle, posterior cranial fossa, and sigmoid sinus worsens prognosis. The invasion of the otic capsule, petrous apex, middle cranial fossa dura, and carotid canal tends to decrease the DSS rate. Specific anatomical invasions, which make margin-negative resection difficult, tend to be an anatomical marker for clinical deterioration.

The superior head of the pterygoid muscle is attached to the anterior disk of the TMJ, and the inferior head of the pterygoid muscle inserts on the mandibular condyle. Our univariate results show that the pterygoid muscle is associated with prognostic factors. Omura et al. (24) reported that TMJ invasion was a prognostic marker because it makes achieving local control difficult. Our study showed that pterygoid muscle invasion, rather than TMJ invasion, was associated with prognosis. In our series, TMJ involvement without pterygoid muscle invasion can be resected with a negative free margin, but more medial tumor invasion can lead to a greater possibility of positive margin resection. Furthermore, if the tumor extends anteriorly to reach the TMJ, this results in invasion of the abundant venous network around the TMJ and pterygoid muscle (pterygoid venous plexus). The invasion of the venous plexus could serve as a route for metastasis spread.

The middle ear invasion upgrades the T stage to T3 according to the modified Pittsburgh classification (1). Middle ear invasion is considered a marker for poor patient prognosis (21). However, some authors have reported that middle ear invasion does not worsen prognosis, and there is a relatively good survival rate for T1–T3 TB-SCC (8, 9). Therefore, it is still uncertain whether middle ear invasion is a prognostic factor. Stell and McCormick (6) suggested it is surprising there was no difference in survival between external and middle ear tumors when considering the tumor origins; prognoses for cases with tumors in the external auditory meatus and middle ear are similar when comparing similar stages of tumors. Manolidis et al. (23) reported the clinical results of 81 cases with temporal bone malignancies. In their study, patients with epithelial malignancies and moderate or total facial paralysis showed a significant survival disadvantage, and anterior tumor spread carried a worse prognosis than middle ear spread, although the numbers were too low to show statistical significance. Tumors involving the middle ear are not necessarily equivalent to a poor outcome (23). Our results support these findings. Interestingly, destruction of the ossicles in the middle ear significantly worsened prognosis, in contrast to middle ear invasion. Therefore, the destruction of the ossicles could be an anatomical prognostic factor that may reflect the molecular and biological characteristics of the tumor responsible for accelerating bone destruction. This finding is new, and further work is needed to conclude whether it is a true anatomical prognostic factor.

Many authors continue to discuss the impact of dural invasion on prognosis (7, 16, 25, 27–29). In 1994, Parasad et al. reported that in cases where dural invasion was present, surgical resection did not improve overall survival after comparing 11 dural invasion cases with resection and nine dural invasion cases without resection. However, they did not adequately study the margins of resection (16). Furthermore, Leonetti et al. (7) mentioned that dura mater and brain invasion represent aggressive biological behavior. Further, some authors reported that dural involvement and intracranial disease did not affect disease-free survival (16, 27, 28). Kawahara et al. (25) reported that of eight cases that had dural invasion suspected preoperatively and who underwent complete resection with a wide safety margin, only one of the eight cases had apparent brain invasion. Dural involvement from preoperative imaging studies did not affect long-term tumor control and survival (25). Seligman et al. (29) argued that dural invasion need not automatically be considered a surgical contraindication in all cases, noting that three of their four cases of TB-SCC survived for over 5 years following STBR. In our study, the invasion of the posterior cranial fossa dura significantly worsened the DSS rate (27.27%), and invasion of the middle cranial fossa dura showed a high HR (HR = 2.846). These results suggest that dural invasion is a prognostic factor. However, if dural invasion can be accurately assessed and *en bloc* negative margin resection achieved, this does not deny the potential for improving the prognosis. However, the number of such cases is considered a limiting factor.

For vascular invasion, invasion of the sigmoid sinus significantly worsened the DSS rate (30.0%), and invasion of the carotid canal showed a high HR in our study (HR = 2.122). Michaels and Wells (30) found tumors penetrating through the bony wall of the middle ear and infiltrating the carotid canal. For extensive infiltration, radical surgical procedures are contraindicated. Parasad et al. (20) reported that two out of four cases showing invasion of the carotid artery died within 2 years, with several authors supporting these findings of poor DSS rates in cases with vascular invasion (20, 31, 32). Based on our findings, we consider that main vessel invasion could be a prognosis factor.

Facial nerve paralysis has been associated with a poor clinical outcome (6, 18, 23), confirming why cases with facial paresis or paralysis are categorized into T4 in the modified Pittsburg classification (1). However, in an analysis of 147 T4 cases from 21 studies comparing cases with and without facial nerve paralysis, Higgins and Antonio (33) concluded there was no significant impact to overall survival and DSS. In our study, facial nerve paralysis was not associated with prognosis. We surmise that our department's policy of aggressive surgical intervention greatly contributed to improved prognoses among facial paralysis cases. Sacrificing the facial nerve leads to a poorer quality of patient life, but aggressive resection of the facial nerve for cases with facial paralysis is worth considering for local control (18).

We found that the prognosis of cases without any invasion of the ossicles, posterior fossa dura, or sigmoid sinus, which were identified as independent markers, was dramatically better. In addition, a greater number of anatomical prognosis factors resulted in poorer patient DSS rates, a difference that was statistically significant. Therefore, both the anatomical site and number of structures affected are thought to be closely related to prognosis. Recently, multidisciplinary collaboration among clinicians in neurosurgery, otorhinolaryngology, and plastic surgery departments within surgical teams, as well as technological advances in surgical equipment and more precise diagnostic imaging, has removed some of the barriers to the challenging negative surgical margin procedure and improved the prognosis of TB-SCC patients. Therefore, classification measures for invasion of anatomical structures will be important in the future.

A staging system reflecting tumor extension that correlates with clinical prognosis is important for understanding prognosis and selecting an appropriate treatment strategy. Establishing the staging system requires accumulating and analyzing large data sets on anatomical tumor extension and prognoses. Applying the new staging system in our study was not possible due to the low sample size. Furthermore, we cannot completely exclude the influence of the treatment modality on our results. Therefore, detailed data sets published from a number of institutions will be necessary to build a large database for this rare cancer.

## Conclusions

Assessing which anatomical structures are susceptible to invasion by tumors may be important for predicting TB-SCC patient prognosis and selecting appropriate treatment planning, especially surgical intervention. In addition to previously reported factors, the destruction of the ossicles in the middle ear cavity can be an anatomical prognosis factor. A large data set with detailed information regarding the extent of the tumor from various institutions will help facilitate future retrospective meta-analyses.

## Data Availability Statement

All datasets presented in this study are included in the article/supplementary material.

## Ethics Statement

The studies involving human participants were reviewed and approved by the ethics review committee of Kyushu University Hospital (#29–43) and the ethics review committee of Fukuoka University Hospital (#2017M091). The patients/participants provided their written informed consent to participate in this study. Written informed consent was obtained from the individual(s) for the publication of any potentially identifiable images or data included in this article.

## Author Contributions

NK and TNa contributed to the study design (patient selection, statistical analyses). NK contributed to writing of the drafts of the manuscript. MM, TNo, NA, and RU contributed to patient inclusion and follow-up. MM, KSat, TH, KK, and RK contributed to collecting the clinical data of patients. NK, TNo, KSag, and AH contributed to data analysis. All authors contributed to critical reading, revision of the manuscript, and approval of the submitted version.

## Conflict of Interest

The authors declare that the research was conducted in the absence of any commercial or financial relationships that could be construed as a potential conflict of interest.
